# Patterns of emergency department utilization by patients on chronic dialysis: A population-based study

**DOI:** 10.1371/journal.pone.0195323

**Published:** 2018-04-17

**Authors:** Paul Komenda, Navdeep Tangri, Evan Klajncar, Amanda Eng, Michelle Di Nella, Brett Hiebert, Trevor Strome, Ricardo Lobato de Faria, James M. Zacharias, Mauro Verrelli, Manish M. Sood, Claudio Rigatto

**Affiliations:** 1 Faculty of Medicine, University of Manitoba, Winnipeg, MB, Canada; 2 Manitoba Renal Program, Winnipeg, MB, Canada; 3 Chronic Disease Innovation Centre, Seven Oaks General Hospital, Winnipeg, MB, Canada; 4 Cardiac Sciences Program, St. Boniface General Hospital, Winnipeg, MB, Canada; 5 Winnipeg Regional Health Authority, Emergency Department Program, Winnipeg, MB, Canada; 6 University of Ottawa, Ottawa, ON, Canada; Postgraduate Medical Institute, INDIA

## Abstract

**Importance:**

Patients on dialysis are often elderly and frail, with multiple comorbid conditions, and are heavy users of Emergency Department (ED) services. However, objective data on the frequency and pattern of ED utilization by dialysis patients are sparse. Such data could identify periods of highest risk for ED visits and inform health systems interventions to mitigate these risks and improve outcomes

**Objective:**

To describe the pattern and frequency of presentation to ER by dialysis patients

**Design:**

Retrospective cohort study using administrative data collected over ten years (2000–2009) in the Province of Manitoba, Canada.

**Setting:**

Patients presenting to any of 9 ED’s in Winnipeg and Brandon Manitoba. These departments serve >90% of the population of Manitoba, Canada (population 1.2 million).

**Participants:**

All patients presenting to an ED in any of 9 emergency departments in Manitoba, Canada.

**Exposure:**

Dialysis status

**Main outcomes:**

Presentation to the ED

**Results:**

Over 2.1 million ED visits by more than 1.2 million non-dialysis patients and 17,782 ED visits by 3257 dialysis patients were included. Dialysis patients presented 8.5 times more frequently to the ED than the general population (age and sex adjusted, p<0.001). For dialysis patients, ED utilization was significantly higher following the long interdialytic interval (33.6% higher Mondays and 19.5% higher Tuesdays vs. other days of the week, p<0.001) and was 10-fold higher in the 7 days before and after the initiation of dialysis.

**Conclusion and relevance:**

The heavy use of ED services by dialysis patients spikes upward following the long interdialytic interval and also in the week before and after dialysis initiation. The relative risks associated with these vulnerable periods were much higher than those reported for clinical patient characteristics. We propose that intrinsic gaps in the structure of care delivery (e.g. 3 times a week dialysis, imperfect surveillance and clinical monitoring of patients with low GFR) may be the fundamental drivers of this periodicity. Strategies to mitigate this excess health risk are needed.

## Introduction

Emergency Department (ED) utilization in North America continues to rise at a rate exceeding population growth [1]. This increase has had a major impact on ED wait times, quality of care, and expenditures worldwide. A small portion of ED users are responsible for a disproportionate utilization of these services compared to the rest of the population [2]. These “frequent users” include individuals of lower socioeconomic status [3, 4], those whose first point of medical contact is the ED [5], and populations with a specific medical condition [3, 6], including patients with kidney failure on dialysis

Dialysis patients tend to be elderly and frail, have multiple comorbid conditions, and frequently require non-dialysis health care services [1–8]. In particular, it is widely assumed that dialysis patients as a population impose a disproportionate burden on emergency departments, contributing to ED congestion and wait times. Although some data exist to support this assertion, with the exception of one large registry report, most studies examining rates of ED utilization among dialysis patients have been limited in scope and methodology.

Importantly, no population-based study has examined the temporal patterns of ED utilization by dialysis patients. A more thorough understanding of the frequency and pattern of ED visits could help health systems appropriately align ED resources with the needs of dialysis patients and facilitate design of pre-emptive health system interventions to reduce ED utilization, improve patient outcomes, and enhance system efficiencies.

In an attempt to fill some of these knowledge gaps, we performed a population based retrospective cohort study linking two large regional databases in order to compare rates of ED presentations in patients both with and without kidney failure on dialysis. We sought to compare temporal and secular trends of ED presentations in these populations, and identify periods of high risk for ED presentation.

## Methods

The study protocol was approved by the Winnipeg Regional Health Authority (WRHA) Research Review Board and the Health Research Ethics Board of the University of Manitoba.

### Study design

We assembled a population-based retrospective cohort of kidney failure and non-kidney failure patients by linking the Manitoba Renal Program (MRP) provincial database, a registry of all Manitoba kidney failure patients, and the Emergency Admission, Discharge, and Transfer (ADT) Database, an administrative registry of all patients presenting to any ED in the Winnipeg Regional Health Authority (WRHA). Linkage was achieved via utilization of unique Personal Health Identifier Number (PHIN).

### Study population

The study population consisted of the entire population within the WRHA, from the period of Jan 1, 2000 to Dec 31, 2009. The WRHA is responsible for the health care of over 700,000 Winnipeg residents and the tertiary care of nearly 500,000 additional residents of Manitoba [9], representing the vast majority of the province’s population.

### Data sources

The MRP registry has tracked both incident and prevalent dialysis patients in Manitoba since 1996. The registry excludes patients with acute kidney injury who required temporary dialysis. For the purposes of the present study, we linked data from Jan 1, 2000 to Dec 31, 2009. The registry is complete for all prevalent kidney failure patients in Manitoba and provides the basis for billing and reporting of provincial dialysis information to the Canadian Organ Replacement Registry (CORR), the body responsible for the collection of medical data of patients with kidney failure in Canada [10]. Data elements captured included the patient’s PHIN, date of initial dialysis treatment, and modality. All changes to vital status and dialysis modality are updated weekly at interprofessional rounds and are reported in the MRP and CORR registries [11].

The ADT system has tracked all ED visits in the WRHA from its inception in 1999 until Dec 31, 2009, with the purpose of providing real-time data entry and patient registration uniformly across the entire region. We linked data from Jan 1, 2000 to Dec 31, 2009. The database is known to have captured 100% of all ED presentations in the WRHA over this period. Data elements recorded include the patient’s PHIN, general demographics, address, time and date of presentation, hospital of presentation, mode of arrival, and Canadian Triage and Acuity Scale (CTAS) score [12]. Data are entered in real time by skilled personnel upon patient presentation to the ED triage nurse.

Data was linked across databases at the individual level via the patient’s PHIN and patients with non-valid or corrupt PHINs were excluded. All linkages were done on a secure server. Prior to exporting the file for analysis, each patient was given a unique study number and the PHINs were purged from the data, ensuring anonymity of the exported file. The resulting linked data file described ED visits in over 1.2 million unique individuals over the study period.

### Variable definitions

#### Dialysis (kidney failure) status

Dialysis status was assessed separately for each ED visit for each patient using the MRP registry. Patients not appearing in the registry were classified as non-dialysis patients for all ED visits. Patients were classified as a dialysis patient if the date of the ED visit for that patient occurred after the date of initial dialysis, as recorded in the MRP registry. Patients who had ED visits before and after initiation of dialysis were classified as non-dialysis patients for ED visits occurring before and as dialysis patients for visits occurring after dialysis start.

#### Dialysis modality

The vast majority of patients on chronic dialysis in Manitoba are treated with in-centre conventional hemodialysis (CHD, 75%) or peritoneal dialysis (PD, 20%), with a small number undergoing home hemodialysis (HHD, 5%). These proportions are similar to other Canadian provinces. For the purposes of the present analyses, we combined CHD and HHD into one group (HD). We classified kidney transplant patients as non-dialysis patients. Over time, patients undergo modality switches such as changing from transplant to dialysis, or HD to PD (and vice versa). Because the MRP registry records the start and end dates of each modality transition, we attributed each ED visit to the concurrent treatment modality.

#### Demographic data

Demographic data were taken from the ADT system. As this data is also in the MRP registry for patients with kidney failure, the data were compared and reconciled in the small number of cases where disagreement existed. Patient age was calculated for each ED visit based on date of birth and date of ED admission.

#### Data analysis

Normally distributed continuous variables of interest were summarized as mean (SD). Dichotomous variables and outcomes were summarized as percentages. *T* tests and ANOVA were used to compare normally distributed measures. The Mann-Whitney *U* test and the Kruskall-Wallis test were used for non-Gaussian distributions. The χ^2^ test was used to compare dichotomous variables.

#### Secular trends in rates of ED visits

For each year of follow-up, a crude yearly ED visit rate for patients on chronic dialysis was calculated as the number of ED visits by dialysis patients in that year divided by the number of dialysis patients registered in the MRP database at the end of that year. Patients who were on dialysis for less than 6 months of the year in question were classified as non-dialysis patients for that year. Because patients on chronic dialysis were older and had a greater proportion of men than the general Manitoba population, we adjusted the crude rates using direct age and gender standardization referenced to the 2006 adult population of Manitoba. Yearly ED visit rates for non-dialysis patients were calculated as the number of ED visits by non-dialysis patients divided by the estimated adult population of Manitoba in that year. The adult population in Manitoba was estimated for each year using linear interpolation of population data from the 2001, 2006, and 2011 censuses.

#### Clustering of ED visits in relation to day of the week

HD patients typically dialyze three times a week on a Monday/Wednesday/Friday or a Tuesday/Thursday/Saturday schedule, and thus experience two short and one long interdialytic interval (i.e. the Friday to Monday interval or Saturday to Tuesday interval, respectively). To address the question of whether ED visits might be clustered on days following the long interdialytic intervals in HD patients, we calculated the rate of ED visits per patient year occurring on each day of the week, stratified by HD, PD, and non-dialysis status. Rates were compared across days of the week and across strata using Poisson regression. The hypothesis that the day of the week effect was more pronounced in HD than in other categories was tested using a formal interaction term (weekday x kidney failure status).

#### Clustering of ED visits in relation to dialysis start date

To address the question of whether ED visits clustered shortly before or shortly after the initiation of chronic dialytic therapy, we calculated the timing in weeks of each ED visit in relation to the dialysis initiation date for each patient (e.g. visit occurred 21–28 days before; 21–28 days after initiation of dialysis). We then calculated the total number of ED visits for each week preceding and each week following initiation of dialysis. We investigated ED utilization up to 52 weeks prior to the initiation of dialysis and 52 weeks after the initiation of dialysis in both HD and PD patients. The weekly frequencies were expressed as rate per patient-year of follow-up.

## Results

Over the study period, the linked dataset included more than 2.1 million ED visits by more than 1.2 million non-dialysis patients and 17,782 ED visits by 3257 dialysis patients. In total, 301 patients had invalid or missing PHINs and were excluded from the study.

**Fig 1:**
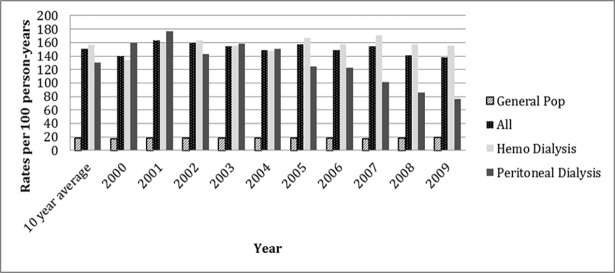
Crude rates of ED visits in dialysis patients vs. the general population.

As demonstrated in Table 1 and Fig 1, the crude rates show a more than eight-fold greater rate of ED presentations by dialysis patients compared to the non-dialysis patients in Manitoba (150 vs. 18 visits per 100 patients per year, p<0.001) over the study period. Age and sex adjusted rates were similar to unadjusted rates. Rates were fairly stable over the ten-year study period, with negligible variation year to year from 2000 to 2009. There was a slightly higher average rate of ED presentation in patients on HD compared to patients on PD (157 vs. 124 visits per patient year, p<0.001) over the study period.

**Table 1:** Dialysis patient visits to the emergency department by year.

Year	General Population (reference)	Dialysis Cohort					
	Crude	All		HD		PD	
	(annual visits/100 patients)	(annual visits/100 patients)		(annual visits/100 patients)		(annual visits/100 patients)	
		Crude	Age/Sex adjusted	Crude	Age/Sex adjusted	Crude	Age/Sex adjusted
**2000**	17.3	140	148	134	144	159	215
**2001**	18.4	163	179	159	175	177	239
**2002**	18.0	159	152	163	157	143	170
**2003**	18.0	155	163	155	166	158	158
**2004**	18.0	149	146	148	148	151	164
**2005**	18.6	157	179	167	187	125	135
**2006**	18.3	149	159	157	167	123	197
**2007**	17.7	155	168	171	188	101	129
**2008**	17.9	141	165	157	184	86.0	98.1
**2009**	19.2	138	149	155	165	76.7	106
**10 year average**	**18.1**	**150**	**153**	**157**	**161**	**124**	**124**

### Temporal trends in ED utilization

**Fig 2:**
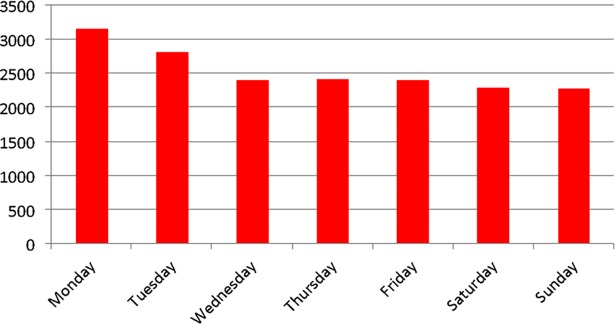
Number of total ED visits by dialysis patients as a function of time and weekday.

In the general population, rates of ED visits were approximately 7% higher on Mondays and 5% higher on Tuesdays compared to the other days of the week. This “post-weekend” effect was much more pronounced in dialysis patients, where rates were 30% higher on Mondays and 15% higher on Tuesdays (Fig 2).

**Fig 3:**
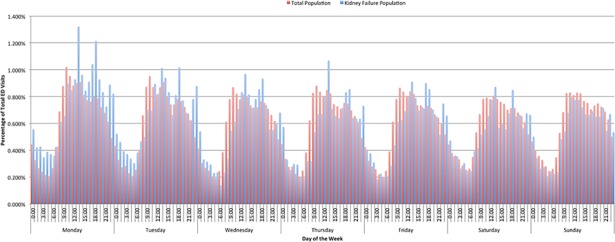
Frequency of ED visits as a function of dialysis initiation.

We also observed a consistent daily trend based on the time of day. In the general population (Fig 3), ED visits showed a relatively bimodal distribution, with a prominent spike in late morning to early afternoon (10:00–15:00) and a slighter peak in the early evening (18:00–20:00). These periods account for approximately 40% of all ED presentations. We also recognized a prominent lull in ED utilization daily in the early morning (1:00–7:00). In contrast, data for the dialysis population (Fig 3) showed generally less distinct diurnal patterns. Of note, a higher proportion of dialysis patients visited the ED in the early mornings of Monday and Tuesday in contrast to this same time in the general population (13.6% vs. 10.4%).

### Relationship of ED presentation to timing of dialysis initiation

**Fig 4:**
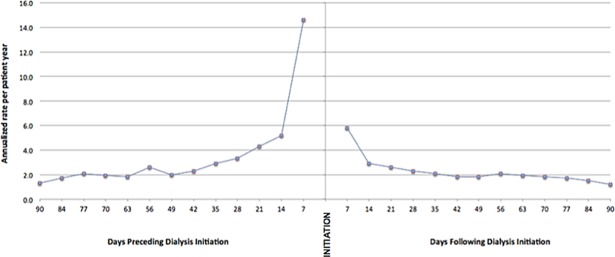
Frequency of ED visits as a function of dialysis initiation.

Among dialysis patients, ED utilization rates differed considerably in the period surrounding dialysis initiation (i.e. time of first dialysis; Fig 4). Several weeks prior to the start of dialysis, rates of ED presentation for incident dialysis patients were found to be higher than that of the general population and of prevalent kidney failure patients. In fact, in the 7 days prior to dialysis initiation, the rate of ED presentation was nine fold higher than the average for prevalent dialysis patients, and nearly 20 fold higher than the general population (14.4 per patient year, p<0.001). In addition, in the 7 days after dialysis initiation there was a four-fold higher rate of ED presentations when compared to the background rate in the average prevalent dialysis patient (5.8 per patient year, p<0.001). When comparing modality type, patients initiating on HD were twice as likely to present to the ED as patients starting on PD.

## Discussion

In this large population-based study, we found that patients with kidney failure on dialysis experienced more than eight-fold higher rates of ED presentation when compared to an age and sex matched cohort from the general population. Importantly, these rates were not homogeneous, but spiked during defined periods of vulnerability; specifically, on Mondays and Tuesdays following a long interdialytic interval, and in the weeks surrounding the initiation of dialysis.

Current literature has long suggested that patients on dialysis use the health care system more frequently than the general population. This utilization includes resources such as specialized clinics, secondary care services [13, 14], and the ICU [8,14,15,16,17]. Several studies have explored “frequent users” of the ED [2,3,18,19,20], but until recently, the identification of kidney failure as a risk factor for ED use has only been established in studies within smaller single-centre populations [2,6,21]. A recently published population based study examined risk factors for ED use in a US Medicare ESRD cohort [22]. The authors found that ED patients presented 6-fold more often to ER than the general population, consistent with the 8-fold rate found in our Canadian population. Both studies thus validate and generalize the observations of smaller studies. Each of these large studies also contributes complementary information about risk. The US cohort contained significant patient level data on comorbidities and hospital admissions. The majority of ED presentations were attributable to dialysis related issues, such as high potassium, volume overload, and bacteremia. The authors were able to identify a large number of risk factors for ED presentation and admission, including sociodemographic factors (younger age, female sex, black race, institutionalization, insurance type) and comorbid medical conditions such as diabetes. While most of these factors had modest impact on risk (i.e. increased or decreased the OR by 3–4%), the presence of catheter hemodialysis access (vs fistula) was associated with a 22% higher odds of ED presentation, identifying this as a potential target for intervention. In contrast, our study focused on the periodicity of ED visits, and showed that specific epochs (after the long interdialytic interval and around initiation of dialysis) were associated with a sharp increase in risk of ED presentation. The effect of this periodicity was marked (30%-900% higher risk), and greater than for most reported sociodemographic and comorbid disease factors.

Importantly these vulnerable periods identify targets for potential health system interventions. Our observation of higher ED risk after the long interdialytic interval is novel and deeply concerning in the context of published data from the United States Renal Data Systems (USRDS) showing a higher mortality rate for dialysis patients on Mondays/Tuesdays compared to the other days of the week [23]. The cause of this “post weekend” effect is thought lie in how in-centre dialysis treatments are structured. In-centre hemodialysis patients typically dialyze three times a week: either Monday-Wednesday-Friday (Mo-We-Fr) or Tuesday-Thursday-Saturday (Tu-Th-Sa). The between-dialysis (interdialytic) interval is 48 hours, except for the Fr to Mo and Sa to Tu intervals, which are 72 hours. It is plausible that the longer 72 hour interdialytic interval may lead to progressive fluid accumulation, more severe electrolyte derangements (e.g. hyperkalemia), and greater cardiovascular instability, resulting in higher need for emergent care or death on the last day (Mo or Tu) of the interval. Whether this higher risk for adversity following the long interdialytic interval applies to all dialysis patients, or is restricted to a subset, is a question of critical importance and will need to be addressed in future research. Importantly, while strategies exist to eliminate the long dialytic interval, such as short daily hemodialysis and home hemodialysis, these strategies have implications in terms of patient care burden (higher with home dialysis) and cost to payers (higher with short daily dialysis). The cost-benefit from patient, societal, and payer perspectives will need to be better understood and balanced before clear recommendations can be made.

The observation of more frequent ED visits around the time of dialysis initiation is also novel, and highlights a period of heightened vulnerability for patients with progressive kidney disease. Some of this spike may be attributable to patients presenting in extremis with undiagnosed CKD who require urgent dialysis (so called “crash starts”). Such patients are often first diagnosed in the ED and plausibly contribute to the excess ED visits observed at or around the time of initiation. However, even patients who were known to have started dialysis electively exhibited a higher rate of ED visits around the time of initiation. For example, the subcohort of patients starting peritoneal dialysis exhibited a similar, albeit attenuated, spike in ED visits around the time of initiation.

It is plausible that patients with advanced CKD approaching the need for dialysis may be at higher risk of ED presentation. Advanced CKD patients are often elderly, have a high prevalence of frailty and mild to moderate cognitive impairment, and multiple comorbid diseases, all of which may be adversely influenced by progressive decline in GFR, leading to higher frequency of ED visits for both medical (e.g. fluid overload/hyperkalemia) reasons, as well as accelerated functional decline (e.g. “failure to thrive”). Conversely, the initiation of dialysis carries new potential risks and complications, including hemodynamic instability, hemodialysis access related complications, and inability to cope functionally with the new burden of dialysis treatment, all of which could contribute to increased visits in the immediate post-initiation period. We hypothesize that population-based programs of CKD surveillance will be needed to decrease the incidence of crash starts due to undiagnosed CKD. In addition, home telemonitoring of nephrology clinic patients with advanced CKD may prevent ED presentations and hospital admissions, as has been shown for heart failure patients [24–29]. Similar research in CKD patients is urgently needed.

Our findings have some direct clinical, health policy, and research implications. First, our data provide health care professionals and policy makers accurate estimates of the impact of dialysis patients on ED utilization at a population level. These data can be used to plan additional ED capacity based on growth of the dialysis population [24].

Second, the excess of ED presentations after the long interdialytic interval, in the context of an identical pattern for death reported by others, highlights the need to carefully examine alternatives to standard, thrice weekly hemodialysis regimens. Further research is needed to address which patients are at greatest risk and to identify the most feasible and cost-effective strategies decrease this risk (e.g. short daily dialysis, home dialysis)

Finally, the spike in ED presentations in the few weeks bracketing the initiation of dialysis needs to be better understood, and strategies to mitigate this risk developed and tested.

The major strength of this study is its population-wide, comprehensive data capture. We analyzed large regional databases that tracked over 1.2 million people attending multiple treatment centers serving nearly the entire population of a Canadian Province. This allowed us to generate population based rates and trends of ED utilization for both dialysis and non-dialysis patients over a decade, and to identify periods of risk for ED visits. To the extent that the population of Manitoba is broadly similar to the larger, multi-ethnic and multi-racial Canadian population, and as the causes and kidney failure are similar to other jurisdictions in North America, results of this study should be applicable to other regions in North America. Our study also has some limitations, many of them implicit in the use of administrative data. The datasets we used were extensive and population based, but limited with respect to individual patient level comorbidity data. Additional research will be required to address patient level risk factors for, and outcomes of, ED presentations.

## Conclusion

The heavy use of ED services by dialysis patients spikes upward following the long interdialytic interval and also in the week before and after dialysis initiation. Further research on strategies to mitigate the excess health risk during these periods of heightened vulnerability is needed. Specifically, research on interventions to better manage or eliminate the risk associated with long interdialytic interval and to better identify and monitor patients around the initiation of dialysis should be prioritized
